# An integrated ultrasound-guided focused ultrasound system enables spatiotemporal control of thermal gene activation in engineered immune cells

**DOI:** 10.7150/thno.118405

**Published:** 2026-01-01

**Authors:** Jeungyoon Lee, Melissa Cadena, Myeongsoo Kim, Seoyoon Song, Ali Zamat, Gabriel A. Kwong, Stanislav Y. Emelianov

**Affiliations:** 1 School of Electrical and Computer Engineering, Georgia Institute of Technology, Atlanta, GA, 30332, USA.; 2 Wallace H. Coulter Department of Biomedical Engineering, Georgia Institute of Technology and Emory University School of Medicine, Atlanta, GA 30332, USA.; 3 Parker H. Petit Institute for Bioengineering and Bioscience, Georgia Institute of Technology, Atlanta, GA, USA.

**Keywords:** focused ultrasound, mild hyperthermia, ultrasound imaging, image-guided hyperthermia, thermal gene switch

## Abstract

**Rationale:** Thermal gene switches (TGSs), engineered into cells, allow controlled gene expression upon heat stimulation, making it a promising tool for therapeutic applications. Their clinical translation, however, has been hindered by the lack of thermal activation platforms that can locally deliver heat and provide safe and accurate temperature control. Existing approaches are limited by poor delivery and localization of heat deep inside the body, reliance on exogenous agents, or the lack of integrated image guidance. To address these challenges, we developed a non-invasive system that combines real-time imaging with mild hyperthermia for reliable and localized activation of TGSs in deep tissue.

**Methods:** We developed a dual-mode ultrasound-guided focused ultrasound (USgFUS) system using a single phased-array imaging transducer for both imaging and heating. The system integrates B-mode imaging and thermal strain imaging (TSI) for real-time anatomical guidance and temperature estimation. We validated the imaging performance both *in vitro* and *in vivo* settings and assessed focused ultrasound (FUS)-induced TGS activation of genetically engineered Jurkat T cells *in vitro* and *in vivo*.

**Results:** The USgFUS system achieved high-resolution and high-contrast B-mode imaging, and it induced localized heating within temperature window of 39-43 °C, consistently within the mild hyperthermia range. TSI accurately estimated temperature elevation during FUS with 0.8 °C mean absolute error. *In vitro*, FUS heating increased transgene expression in TGS-engineered Jurkat T cells by ~150-fold compared to unheated controls, with negligible viability loss. *In vivo*, USgFUS selectively activated TGS in tumor-bearing mice, yielding a significant increase in transgene expression compared to unheated controls.

**Conclusion:** This study introduces a dual-mode USgFUS system designed for non-invasive TGS activation. The system integrates local mild hyperthermia with real-time anatomical guidance and temperature monitoring using a standard clinical imaging probe. The results collectively demonstrate strong performance in preclinical models and engineered cells, enabling safe, spatiotemporally precise thermal gene regulation. Ultimately, our platform provides a foundation for future advancements in gene therapy, immunomodulation, and other biomedical applications.

## Introduction

Mild hyperthermia (39-43 °C) is a well-established therapeutic tool for biomedical applications ranging from pain relief to cancer therapy and immunomodulation 1-3. Recent advances in synthetic biology have further amplified its potential through thermal gene switches (TGSs), which can precisely regulate transcriptional activity in response to mild hyperthermia from external stimulation 4, 5. TGSs enable safe, tunable, and reversible gene expression, opening new possibilities for targeted drug delivery, gene therapy, and cellular therapies 6-8. However, the safety and efficacy of TGS-based interventions depend on accurate heat control 6, 9. Excessive heat can cause irreversible tissue damage, whereas insufficient heating may lead to limited activation level. Therefore, precise thermal control is essential to fully harness the therapeutic potential of TGS. Several TGS activation strategies have been explored, including photothermal therapy (PTT) with infrared imaging 9-12, magnetic hyperthermia with optical fiber temperature sensor 13-15, and focused ultrasound (FUS) with magnetic resonance (MR) thermometry 16-20. However, PTT is limited by shallow penetration and risks of surface overheating 21, while magnetic hyperthermia suffers from uneven heating and high costs 22. Both methods also rely on exogenous agents, raising concerns about spatial precision and biocompatibility 23. By contrast, FUS offers a non-invasive approach with deep tissue penetration up to tens of centimeters and submillimeter spatial precision 24-26, making it a highly promising modality for controlled thermal gene activation.

Effective FUS-mediated hyperthermia relies on image guidance, with two critical aspects: anatomical visualization and temperature monitoring. While MR imaging is widely used 27, 28, its high cost, slow frame rate, limited portability, and integration challenges with FUS hardware limit its practicality for applications requiring repeated imaging or resistance to physiological motion. By contrast, ultrasound (US) imaging provides a cost-effective, real-time alternative that integrates seamlessly with FUS systems 29-31. US B-mode imaging offers high-resolution tissue visualization, aiding treatment planning that ensures the safety of vulnerable anatomic structures. Among various US-based thermometry methods, thermal strain imaging (TSI) has been studied most extensively for monitoring temperatures in the mild hyperthermic range based on the temperature-dependent sound speed change 30-33.

Several US-guided FUS (USgFUS) systems have been developed for localized hyperthermia under image guidance. The most common configuration pairs a single element or phased-array therapeutic transducer and a separate diagnostic imaging array 34-38. Having dedicated systems for each function, this setup optimizes acoustic parameters for treatment and imaging, enhancing overall performance. However, these systems are often complex and bulky, and the spatial separation of transducers can lead to misalignment, potentially causing targeting errors. An alternative approach integrates therapeutic and monitoring functions into a single dual-mode phased-array transducer, allowing seamless switching between treatment and imaging 39-43. This configuration offers inherent spatial registration, compactness, and improved control. However, because FUS transducers and diagnostic imaging arrays have distinct piezoelectric properties, using one transducer for both functions may compromise performance, requiring careful system optimization towards specific applications 44-46.

In this study, we present an integrated dual-mode USgFUS system designed for precise spatiotemporal regulation of TGS activation. This system combines FUS for localized hyperthermia with real-time imaging, providing anatomical guidance and temperature monitoring. Employing a single phased-array imaging transducer, this design ensured spatial alignment between treatment and monitoring planes while minimizing system complexity. We validated the system's capabilities for high-resolution imaging, robust temperature estimation, and effective TGS activation both *in vitro* and *in vivo*. Our results support USgFUS as a cost-effective, real-time solution for TGS activation, utilizing a phased-array imaging transducer already widely used in research and clinical settings. This work establishes a non-invasive and efficient platform for thermal gene modulation, with broad implications for gene therapy, immunomodulation, and other biomedical applications requiring spatiotemporally controlled induction of mild hyperthermia.

## Results and Discussion

**Figure 1:**
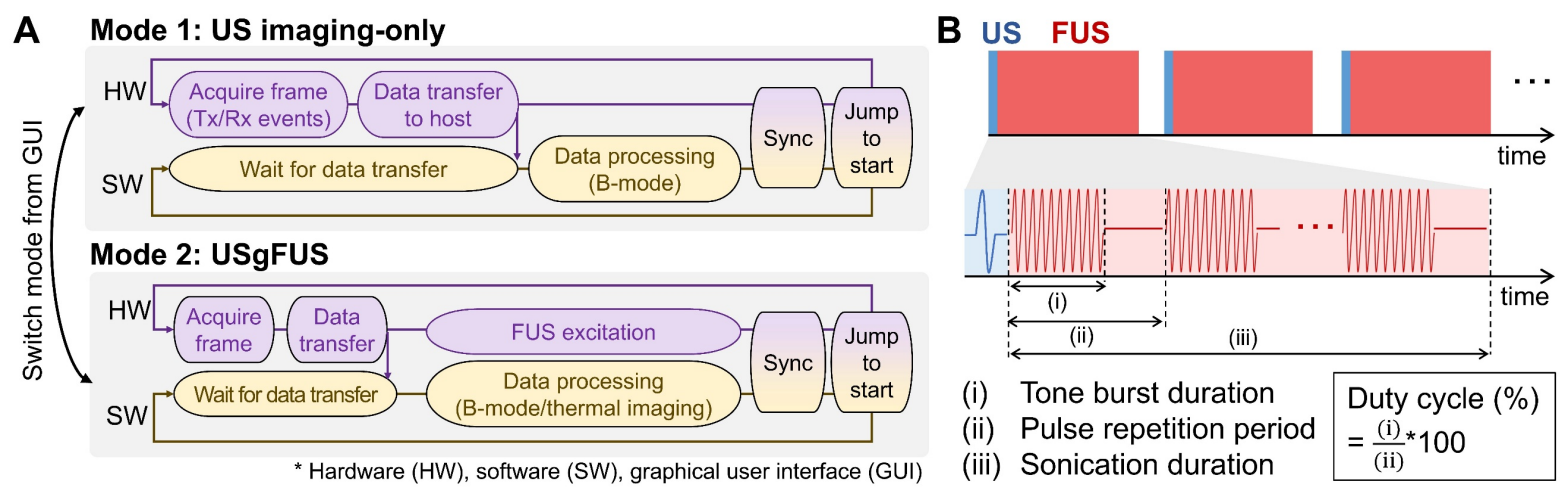
** Integrated ultrasound-guided focused ultrasound (USgFUS) system design.** (A) The system operational sequence is designed to switch between ultrasound (US) imaging-only and USgFUS modes from graphical user interface (GUI). (B) Schematics of alternating US imaging and focused ultrasound (FUS) excitation and definitions of associated acoustic parameters.

We developed an integrated USgFUS system that switches between US imaging-only and USgFUS modes using a single phased-array transducer (Figure 1A). A graphical user interface (GUI) allows real-time mode switching and acoustic parameter adjustments for both imaging and heating. This enables users to first localize the target using US B-mode imaging, then initiate treatment under real-time guidance. Since the identical phased-array transducer is shared for both imaging and heating, seamless switching is possible without misalignment concerns.

To prevent image artifacts caused by acoustic interference, imaging and heating pulses were interleaved during USgFUS mode (Figure 1B). Each image acquisition was followed by multiple pulsed-wave FUS excitations, with user-defined burst duration, pulse repetition period, and sonication duration. The duty cycle, calculated as (burst duration)/(pulse repetition period)×100 (%), along with output voltage amplitude, determines the FUS acoustic power 24, 47. Once in sonication duration, updated B-mode frames offer real-time visualization of the treatment area. This cycle continues until treatment is stopped or imaging-only mode is resumed.

While imaging and FUS share the same system resources, distinct configurations were determined to optimize each function. For imaging, the full 128-element aperture produced short imaging pulses at 7 MHz center frequency, achieving high spatial resolution and a wide field of view 48. For heating, only the central 78 elements were used for burst excitation, with phase delays applied to generate a focal spot at 10 mm depth along the centerline. A lower center frequency of 4.8 MHz was selected - near the lower limit of the transducer's bandwidth - to reduce beam distortion and minimize off-target acoustic absorption 47.

**Figure 2:**
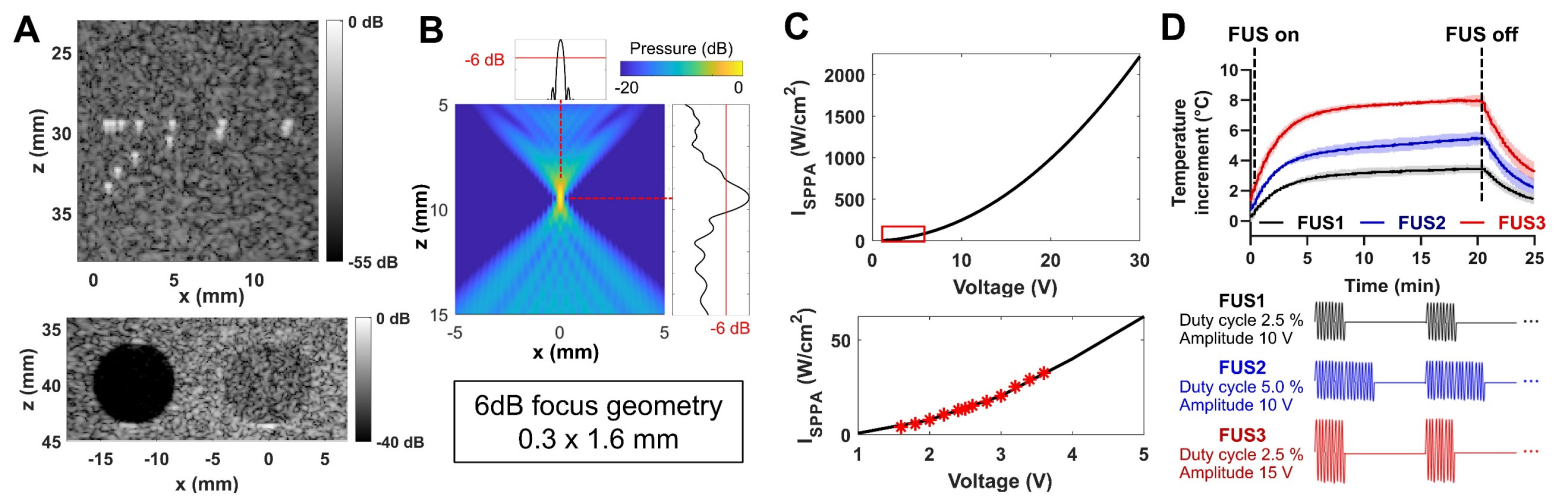
** Imaging and heating performance of the USgFUS system.** (A) B-mode images of a commercial phantom point targets (top) and cyst inclusions (bottom). (B) A simulated FUS beam pattern of the imaging array showed a 6 dB focal size of 0.3×1.6 mm. (C) Acoustic intensity at the focus, measured with a hydrophone within the operational range and extrapolated to the USgFUS system's maximum output voltage. (D) Temperature profile of FUS heating of 360 μL water with varying duty cycles and amplitudes (n = 3, data are presented as mean ± s.d.).

Imaging performance was tested using a general-purpose ultrasound phantom 49. Plane wave B-mode imaging resolved 0.5 mm-spaced point targets, achieving a 0.4 mm lateral full-width half-maximum (FWHM) resolution at a depth of 30 mm (Figure 2A). In the same phantom, contrast-to-noise and contrast ratios for anechoic and -3 dB hypoechoic cysts were 12.3 dB and 6.4 dB, respectively, at a depth of 40 mm (Figure 2A). The identical plane wave imaging configuration was used in subsequent phantom and animal experiments.

FUS heating was evaluated through simulations and experiments. Beam field simulations showed a 6 dB focal geometry of 0.3×1.6 mm at 10 mm depth (Figure 2B). Hydrophone measurements estimated the spatial peak pulse average intensity (I_SPPA_) of approximately 2200 W/cm^2^ at focus with 30 V output voltage, corresponding to the maximum capacity of the USgFUS system using 78 elements in burst mode (Figure 2C). With varying duty cycles and output voltage, FUS raised water temperature in a 96-well plate by 3.4-8.0 °C within 20 min (Figure 2D). The temperature rose rapidly at the beginning, reaching 80% of the peak elevation within 5 min, followed by slower logarithmic increase. These results confirm the USgFUS system's capability for mild hyperthermia and provide foundations of acoustic parameter adjustments for heating.

**Figure 3:**
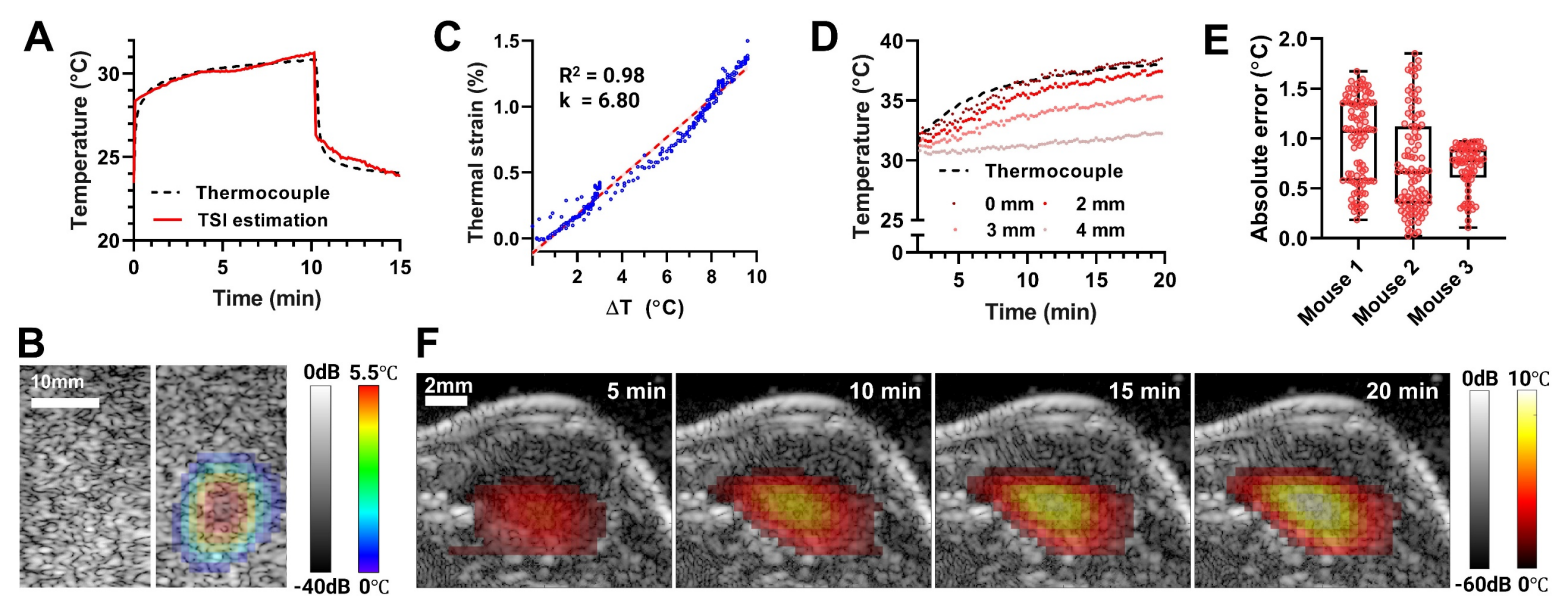
** US thermal strain imaging (TSI) during FUS-induced mild hyperthermia.** (A) Temperature profile at FUS focus in a tissue-mimicking phantom, measured by a thermocouple and TSI. (B) Overlaid B-mode and TSI images of the phantom. (C) *In vivo* thermal strain calibration of mouse tumors using thermocouple measurements. (D) Temperature changes in tumor estimated by TSI at 0, 2, 4 mm distance from FUS focus, compared to reference thermocouple measurements at the focus. (E) Absolute error of TSI temperature estimation relative to thermocouple measurements in three different mouse tumors (all data points are shown with min-max range and median bar). (F) Overlaid B-mode and TSI images of a mouse tumor at different time points.

Image-guided mild hyperthermia was demonstrated in phantoms and tumor-bearing mice. In a tissue-mimicking phantom, FUS-induced temperature elevation was recorded using a thermocouple inserted at the focal point (Figure 3A). Within a minute, the temperature increased by 5.5 °C, entering the mild hyperthermia range, assuming an average body temperature of 36.5 °C. Continued FUS sonication for 10 min increased the temperature by 7.4 °C. Thermal strains were calculated from B-mode images acquired during FUS application, calibrated using the thermocouple temperature data, then converted to temperature maps (Figure 3B). TSI was able to estimate the temperature at focus with mean absolute error (MAE) of 0.25 °C compared to the thermocouple reference (Figure 3A).

We further validated the system's capabilities *in vivo* in preclinical murine tumor models. The tissue-dependent thermal strain coefficient (k) was calibrated across three biologically independent tumor samples. Linear regression between thermocouple measurements and thermal strain over 10 °C increment resulted in k = 6.80 with a strong correlation (R^2^ = 0.98, Figure 3C). This calibration result was then applied to TSI for three additional tumor-bearing mice. Figure 3D shows representative TSI-based temperature estimation during FUS heating at 0, 2, 3, and 4 mm from FUS focus, along with thermocouple readings at the focus included for validation. The MAE between TSI and thermocouple measurements at focus was 0.8 °C, with a maximum absolute error of 1.85 °C (Figure 3E). TSI-based temperature at increasing distance confirmed that heat generated within the submillimeter FUS focus diffused into adjacent tissue, producing progressively reduced maximum temperatures (88.6%, 66.6%, and 35.6% of the focal temperature, at 2, 3, and 4 mm, respectively) and slower heating rates (90.0%, 63.2%, and 24.9% of the focal rate, respectively). Reconstructed 2D temperature maps over time demonstrated a peak focal temperature increase of 10 °C, with gradual heat diffusion into surrounding tumor tissue (Figure 3F). These results confirm the system's ability to monitor and deliver targeted thermal therapy and establish the calibrated k coefficient for subsequent *in vivo* thermal gene activation.

Heat accumulation and diffusion can vary considerably across individual subjects *in vivo* due to focal geometry distortion, tissue heterogeneity, and vascular perfusion. The 2D temperature maps generated by our USgFUS system provide real-time information on heat intensity and duration, inherently capturing such *in situ* effects. With this information, the system can better control the treatment zone and avoid overheating, by dynamic adjustments of acoustic parameters, focal steering, and termination timing. Collectively, these capabilities highlight the ability of USgFUS system to deliver safe, well-controlled thermal therapies *in vivo*. Note that integration with non-invasive vascular imaging methods (e.g., Doppler ultrasound) could further enhance treatment planning and monitoring, and our system is readily compatible with such modalities.

**Figure 4:**
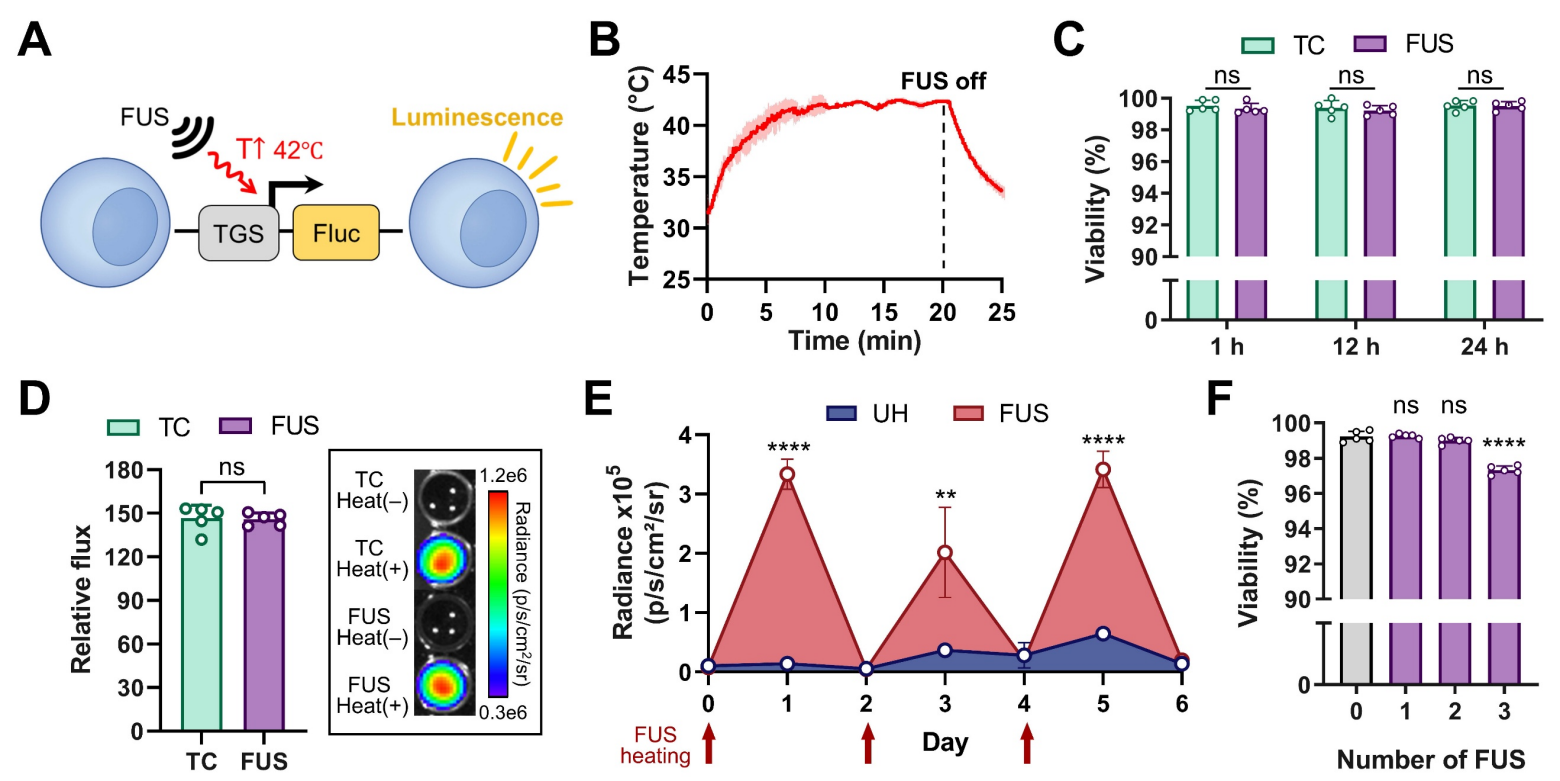
** FUS-mediated thermal gene switch (TGS) activation *in vitro*.** (A) Jurkat T cells were engineered to express firefly luciferase (Fluc) upon TGS activation at 42 ℃ (TGS.Fluc Jurkat) 9. (B) Temperature profile of FUS heating in a 96-well plate, measured by a thermocouple (42 ℃ for 20 min). (C) Cell viability measured 1, 12, and 24 h post-heat using a thermocycler (TC) and FUS. (D) Representative IVIS image and luminescent signal 12 h post-heat using TC and FUS relative to unheated (UH) controls. (E) Luminescence signal measured 12 h after each of three repeated FUS heating sessions performed every other day over 6 days, compared with UH controls. (F) Cell viability measured 12 h after 1-3 FUS heating sessions, compared with UH (0 session) controls. n = 5, error bars = s.d., ns = non-significant, ** p < 0.01, **** p < 0.0001, two-tailed t test.

The USgFUS system was next used to activate thermo-inducible gene expression *in vitro* using TGS-engineered Jurkat T cells. These cells were engineered to express firefly luciferase (Fluc) upon TGS activation (TGS.Fluc Jurkat, Figure 4A) 9, 10. FUS stimulation was applied for 20 min to maintain the cells at 42 °C, following a previously established heat treatment protocol (Figure 4B) 9. Thermocycler heating was used as a gold standard for comparison of cell viability and thermal activation efficacy. All experiments were performed with five independent biological replicates. Cell viability was assessed at 1, 12, and 24 h post-heating to capture both immediate and delayed effects (Figure 4C). At every time point, viability loss was negligible (< 1%) with no significant difference between thermocycler- or FUS-heated groups (p > 0.4). Luminescence measurements taken 12 h post-heat treatment showed a ~150-fold increase in both thermocycler- and FUS-treated groups relative to unheated controls, with no significant difference between the two heated conditions (Figure 4D, left panel). IVIS bioluminescence imaging further confirmed the difference between heated and unheated conditions (Figure 4D, right panel). In addition, we evaluated the long-term effects of FUS heating by delivering three heating sessions every other day over a 6-day period and measuring luminescence and cell viability 12 h after each session. Each session comprised five independent FUS heating experiments to ensure measurement robustness. Luminescence increased significantly after each FUS treatment and returned to baseline between sessions, while viability loss remained minimal (< 3%) at the end of the final session. These findings indicate that the thermal responsiveness and viability of TGS-engineered cells are preserved under repeated FUS delivery. Together, these results demonstrate that the USgFUS system can reliably activate TGS *in vitro* with high efficiency while maintaining cell viability. This establishes a strong foundation for subsequent *in vivo* applications of USgFUS-mediated gene regulation.

**Figure 5:**
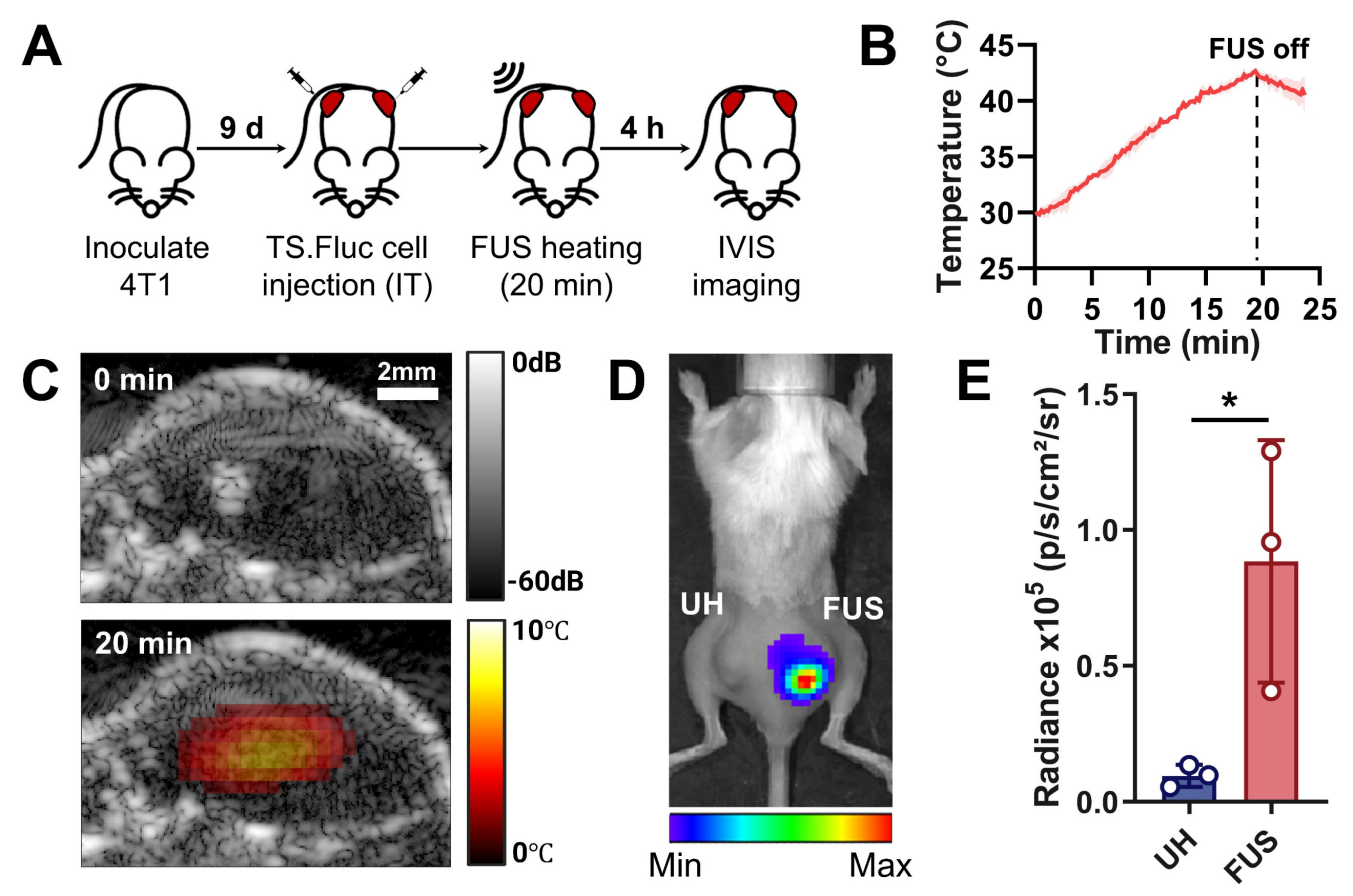
** Local TGS activation via USgFUS *in vivo*.** (A) Timeline of the *in vivo* experiment using bilateral tumor-bearing mice (IT: intratumoral). (B) Temperature profile at the FUS focus, estimated from TSI (42 ℃ for 20 min). (C) Overlaid B-mode and TSI images before (top) and at peak heating (bottom). (D) Representative IVIS image and (E) quantified luminescent signal of UH and FUS-heated tumors 4 h post-heating. n = 3, error bars = s.d., * p < 0.05, two-tailed t test.

To demonstrate spatiotemporal control of thermosensitive gene expression using the system *in vivo*, TGS.Fluc Jurkats were injected intratumorally into bilateral flank tumors in BALB/c mice, with only one side receiving FUS stimulation (Figure 5A). This experimental design allows us to decouple the effect of systemic immune cell delivery from the system's ability to induce spatiotemporally confined heating. During FUS treatment, temperature elevation was monitored using TSI reconstructed with the previously calibrated k coefficient (Figure 5B). TSI-based temperature estimation at the focal spot successfully reached the activation threshold (40-42 °C), assuming an initial tumor temperature as ~30 °C, which was given by the average initial temperature recorded in previous *in vivo* heating experiments. Real-time B-mode imaging provided anatomical visualization of the tumor, while overlaid TSI displayed the temperature distribution within the tissue (Figure 5C). The combination of these two imaging modalities allows accurate spatial localization of the target tissue and control of heat duration and intensity, ensuring safe and effective gene activation. IVIS imaging performed 4 h after heat treatment revealed localized luminescence from TGS activation exclusively in the FUS-treated tumor compared to the unheated control on the opposite side (Figure 5D). Quantitative analysis confirmed significantly increased luminescence in FUS-treated tumors (Figure 5E).

While the current results demonstrate robust TGS activation, further improvements in activation efficiency are anticipated with ongoing advancements in USgFUS technology, particularly through the development of a real-time feedback loop for continuous monitoring and adaptive control 29. Electronic steering of the FUS focal spot could enable heating at multiple locations, expanding the treated area 50. Integration with complementary functional imaging modalities such as photoacoustic or bioluminescence imaging could further optimize heat distribution and treatment accuracy 17, 51, 52. Collectively, these enhancements will refine the USgFUS system's spatiotemporal precision for non-invasive thermal gene activation and broaden its therapeutic applications.

Our current study used intratumoral injection of engineered immune cells, which is widely recognized, both clinically and preclinically, as a strategy to enhance safety, bypass homing barriers, and rigorously evaluate controllable cell therapies 53-55. However, validation of our system with systemic administration of TGS-engineered cells will further broaden the impact of the platform, as intravenous delivery is the standard route in many immunotherapies, including adoptive T cell transfer and chimeric antigen receptor (CAR)-T cell therapy 56, 57. Importantly, systemic delivery combined with localized thermal control has already been demonstrated using photothermal heating 10, supporting the broader translational potential of our approach and motivating future studies exploring intravenous delivery of TGS-engineered cells.

In this study, we intentionally excluded therapeutic constructs and instead used luciferase as a neutral reporter to avoid confounding systemic effects, thereby establishing a clear proof-of-concept. Moving forward, incorporating therapeutic constructs such as cytokines, TNF-related apoptosis-inducing ligand (TRAIL), or CAR receptors and evaluating functional outcomes (e.g., tumor growth inhibition, immune activation) will be essential for translational applications. Prior work has already shown that TGSs can regulate therapeutic constructs under localized heating - for example, photothermal activation of CAR-T cells engineered to secrete IL-15 superagonists and bispecific T cell engagers (BiTEs) produced localized antitumor responses 10, and FUS-mediated control of CAR-T cells enabled spatially confined activation and tumor regression 16. These findings underscore that our USgFUS system, now validated for heat-gated reporter protein expression, is directly compatible with therapeutic applications and well positioned for future translation.

## Conclusions

This study demonstrated the development and validation of an integrated dual-mode USgFUS system that enables image-guided regulation of TGS activation through non-invasive mild hyperthermia. By utilizing a single phased-array imaging transducer for both imaging and heating, our system achieves seamless spatial registration, compact design, and streamlined operation without compromising performance. Interleaved imaging and pulsed-wave FUS sequences allow for simultaneous anatomical guidance and thermal monitoring, with customizable acoustic parameters enabling controlled and localized heating.

Comprehensive *in vitro* and *in vivo* validations confirmed the system's ability to generate mild hyperthermia within the temperature window (39-43 °C), with TSI providing accurate estimation of temperature distribution (MAE = 0.8 °C) overlayed on B-mode images showing the anatomical background. Successful activation of TGS in engineered Jurkat T cells was demonstrated both *in vitro* and in mouse tumor models, without affecting the cell viability and high spatial specificity of gene expression. Longitudinal studies further confirmed that thermal responsiveness and viability of engineered cells are preserved under repeated FUS delivery. These findings underscore the system's potential for spatiotemporally precise heat delivery for thermal-sensitive gene regulation.

Looking forward, future integration of real-time thermal feedback control, electronic beam steering for multifocal heating, and complementary imaging modalities (e.g., photoacoustic or Doppler imaging) could further enhance treatment accuracy and therapeutic efficacy. Furthermore, incorporating therapeutic constructs and conducting functional validation assays will be essential for clinical translation. Prior studies have already demonstrated that TGSs can regulate therapeutic constructs under localized heating, and our platform provides the comparable spatiotemporally gated control validated here with a protein reporter.

Overall, this work establishes a cost-effective, versatile, and clinically accessible platform for spatiotemporally controlled induction of mild hyperthermia, paving the way for next-generation therapies involving remote cellular interventions.

## Materials and Methods

### Phantom fabrication

All materials were purchased from Sigma-Aldrich and used as received, unless otherwise stated. Agarose-milk phantoms were prepared following a previously established protocol 58. Fat-free milk, Dulbecco's Phosphate Buffered Saline, and agarose powder were combined and microwaved to 90 °C until completely dissolved. The molten solution was degassed for 5 min, stirred for 10 min at 90 °C while adding silica gel, then degassed for another 5 min. After adding n-propanol, stirring continued until the solution cooled to 40 °C, at which point the solution was poured into a 55×55×70 mm mold and left to solidify overnight at room temperature.

### Cell culture

In all cases, cells were cultured in RPMI-1640 supplemented with 10% fetal bovine serum and 1% penicillin/streptomycin, refreshed every 2-3 days. 4T1 cells were passaged at ~80% confluence. For passaging, cells were detached using 0.05% Trypsin-EDTA (Corning Inc.) for 3 min, neutralized with complete medium, and washed by centrifugation at 125 g for 5 min, then subcultured at a 1:10 ratio. Jurkat T cells were subcultured every 2-3 days to maintain the density between 1×10^5^ to 1×10^6^ cells/mL. Cells were incubated at 37 °C in a 5% CO_2_ humidified incubator (Fisher Scientific).

### Animal model

BALB/c mice (4-6 weeks old, The Jackson Laboratory) were housed under Georgia Tech Division of Animal Resources guidelines, with all animal protocols approved by the Georgia Tech Institutional Animal Care and Use Committee (protocol no. A100281) and all relevant ethical regulations followed. Each mouse received subcutaneous injections of 4T1 breast cancer cells into both flanks (5×10^6^ cells in 50 μL saline). Experiments began on day 9 post tumor inoculation when the average tumor volume reached 120 mm^3^ (0.5×W^2^×H).

### USgFUS system

The USgFUS system consisted of a Vantage 128 ultrasound research platform (Verasonics Inc.), a 128-element linear transducer (ATL L7-4, Philips Healthcare), and a host controller PC. The system alternated between imaging and heating functions, sharing all the hardware resources including the transducer. Internally, a separate power supply with increased capacity was dedicated to heating for extended burst excitation. Overall system control was managed via a custom GUI-based software program on the host PC.

### B-mode imaging

B-mode images were continuously acquired in US imaging-only mode, while 10 frames were collected every 11 s during FUS-off interlude in USgFUS mode. B-mode imaging used plane wave compounding method (21 angles, -18° to +18°) at 190 Hz framerate 59, 60. Beamforming, post-processing, and display update on GUI for real-time imaging were conducted using Vantage system's embedded functions, then evaluated using a general-purpose ultrasound phantom (054GS GP, CIRS). Beamformed B-mode frames acquired during USgFUS mode were saved and further processed for thermal imaging.

### TSI processing

TSI was performed during USgFUS mode by selecting the most stationary B-mode frame in 10-frame set at each FUS-off interval, computing pixel displacement vectors between consecutive time points (~10 s interval), and deriving thermal strain by taking the axial gradient of the displacements 61-63. Thermal strain was accumulated in time, then converted into temperature change maps by multiplying an experimentally calibrated thermal strain coefficient (k).

The coefficient k is a tissue-dependent constant required to convert thermal strain into a temperature map 31, 32. In this study, the thermal strain coefficient for mouse tumors was determined via linear regression between reference thermocouple (K type, 0.003″ Dia., Omega Engineering) readings and computed thermal strain. The thermocouple was inserted into tumor tissue during USgFUS heating, and strain values were averaged from a 0.5×0.5 mm region of interest (ROI) around the thermocouple location. A total of 284 datapoints from three biologically independent samples over 10 °C of temperature change was used in the calibration.

### FUS beamforming and acoustic measurements

The FUS acoustic field was simulated using Field II program 64, 65, configuring transducer parameters for creating an acoustic focus at 10 mm depth along the centerline. A 2.0 mm needle type hydrophone (Precision Acoustics) was used to measure acoustic intensity in a water tank, with the hydrophone scanned in three dimensions for focal spot localization. The measurement was conducted within the operational safety limit of the hydrophone and linearly extrapolated to the maximum output voltage of the USgFUS system. To assess heating profiles, FUS was applied from above to a 96-well plate filled with 360 μL water per well. Temperature changes were recorded at 1 Hz using a thermocouple placed inside the well.

### *In vitro* thermal activation and assays

Jurkat T cells engineered to express the Fluc in response to mild hyperthermia (40-42 °C) 9, 10 were prepared at 5-10×10^6^ cells/mL in complete cell culture media. For thermal activation, cells were heated for 20 min at 42 °C using either a thermocycler (C1000, Bio-Rad) or the USgFUS system. For FUS heating, cells were seeded in 96-well plates, each well containing ~2-4×10^6^ cells. FUS transducer was positioned above the top opening of the well with acoustic coupling using a water-filled coupling cone and a thin membrane (Sonic Concepts Inc.), and a thermocouple was placed inside the well to monitor the temperature.

After heating, cells were incubated at 37 °C and 5% CO_2_. Viability was assessed 1 h, 12 h and 24 h after the termination of heating via Zombie NIR staining (BioLegend) and flow cytometry (Aurora, Cytek Biosciences), then analyzed with FlowJo (FlowJo LLC). Fluc activity was quantified 12 h post-heating using luciferase assay buffer prepared according to the manufacturer's instructions (Gold Bio) and bioluminescence imaging system (Xenogen IVIS, Caliper Life Sciences). Luminescence was measured as the average radiance (photons/s/cm²/sr) within the ROIs established for each well using the Living Image software package (PerkinElmer).

For repeated FUS heating experiments, the remaining cells after viability and luminescence assays were incubated for one additional day, resuspended to the same concentration as in the first FUS session, then subjected to the same heating and assay procedures.

### *In vivo* thermal activation and bioluminescence imaging

Engineered Jurkat T cells (5×10^5^ cells in 50 μL saline) were injected intratumorally into each tumor. Under anesthetized with isoflurane, tumor sites were heated using the USgFUS system with 2.5% duty cycle and ~1000 W/cm^2^ I_SPPA_ for 20 min. To assess Fluc activity, luciferin was injected intraperitoneally (200 μL, 15 mg/mL) following established protocol from the manufacturer 66 4 h post-heating, and mice were imaged via IVIS every 10 min for 1 h. Integration time was set to automatic, ROIs around the whole tumor were defined within the Living Image software package and luminescence was quantified as average radiance (photons/s/cm^2^/sr).

### Software and statistical analysis

Ultrasound image processing was performed in MATLAB (MathWorks). Statistical analysis was conducted in GraphPad Prism 8 (GraphPad Software), with results reported as mean ± s.d. and significance was set as *p < 0.05, **p < 0.01, ***p < 0.001, ****p < 0.0001.

## Abbreviations

BiTE: bispecific T cell engager
CAR: chimeric antigen receptor
Fluc: firefly luciferase
FUS: focused ultrasound
FWHM: full-width half-maximum
GUI: graphical user interface
I_SPPA_: spatial peak pulse average intensity
IT: intratumoral
MAE: mean absolute error
MR: magnetic resonance
PTT: photothermal therapy
ROI: region of interest
TC: thermocycler
TGS: thermal gene switch
TRAIL: TNF-related apoptosis-inducing ligand
TSI: thermal strain imaging
UH: unheated
US: ultrasound
USgFUS: ultrasound-guided focused ultrasound
